# Editorial: Special Issue “Stem Cell Biology and Cancer”

**DOI:** 10.3390/ijms241411533

**Published:** 2023-07-16

**Authors:** Carolina Vicente-Dueñas, Isidro Sánchez-García, Geoffrey Brown

**Affiliations:** 1 Institute for Biomedical Research of Salamanca (IBSAL), Department of Pediatrics, Hospital Universitario de Salamanca, Paseo de San Vicente, 58-182, 37007 Salamanca, Spain; 2 Experimental Therapeutics and Translational Oncology Program, Instituto de Biología Molecular y Celular del Cáncer, CSIC/Universidad de Salamanca, Campus Miguel de Unamuno s/n, 37007 Salamanca, Spain; 3 Institute for Biomedical Research of Salamanca (IBSAL), Campus Miguel de Unamuno s/n, 37007 Salamanca, Spain; 4 School of Biomedical Sciences, Institute of Clinical Sciences, College of Medical and Dental Sciences, University of Birmingham, Edgbaston, Birmingham B15 2TT, UK; g.brown@bham.ac.uk

Cancer stem cells (CSCs) are now well-established as key players in tumor initiation, progression, and therapy resistance. Understanding the mechanisms underlying CSC generation, maintenance, and developing targeted therapies against them is crucial to improving cancer treatment outcomes. CSCs are suggested to be responsible for cancer relapse and drug resistance due to their ability to self-renew and differentiate into a heterogeneous population of cancer cells. Conventional chemotherapeutics and radiotherapy are often very effective against the bulk cells of cancer, which proliferate, but they forgive CSCs that are responsible for disease relapse. In this regard, therapeutics that specifically target CSCs may provide an effective cure for cancer.

The Special Issue, entitled “Stem Cell Biology and Cancer”, of the *International Journal of Molecular Sciences* includes a total of eight contributions that shed light on the field of CSC research and highlights some potential therapeutic strategies.

The glioblastoma stem cells (GSCs) are an example of CSC, and most standard treatments fail to completely eradicate them, causing disease recurrence. GSCs seem to represent the main reason for the low efficacy of cancer therapy and for the short relapse time in patients with glioblastoma, a highly malignant brain cancer. Rodriguez et al. [1] focus on GSCs and their role in the aggressive nature and therapy-resistance of glioblastoma. GSCs’ resistance to radio- and chemotherapy could be explained by their high capacity for extensive DNA repair, quiescence, higher mitochondrial reserve, and localization in the hypoxic niche. The study highlights the need to unravel the heterogeneity of GSCs in order to develop effective therapeutic approaches. The authors emphasize the activation of specific signaling pathways and epithelial–mesenchymal transition in glioblastoma-initiating stem cells, leading to the development of a metastatic phenotype. Overcoming therapeutic challenges in glioblastoma is critical for improving prognosis and patient survival. Further research is needed to better understand the molecular and cellular characteristics of GSCs and to develop targeted therapies that are specifically designed to eliminate these cells.

Leukemia stem cells (LSCs) are one of the most-studied CSCs to date. Unlike hematopoietic stem cells (HSC), the offspring of LSC, which often arise from the transformation of a HSC and sustain the hierarchy of cells for leukemia, belong to just one cell lineage. “The Social Norm of Hematopoietic Stem Cells and Dysregulation in Leukemia” [2] explores the social behavior of HSC and the dysregulation observed in leukemia. The author discusses the complex ecosystem of the hematopoietic cell system, which is highly adaptive, and able to meet the steady-state and emergency needs of mature blood cell production. The article highlights the social tailoring of HSC to generate lineage-biased cells while ensuring it remains versatile and capable of adopting different pathways. In contrast, LSCs disrupt the normal cellular society by restricting their offspring to a single cell lineage. The study also discusses how some oncogenes set the fate of LSCs to a single lineage. Additionally, the destructive and altering effects of leukemia cells on bone marrow stromal niches is examined, as well as leukemia cells’ ability to create their own niches. These antisocial behaviors contribute to the pathogenesis of leukemia. A clear example of this scenario is chronic myeloid leukemia (CML). In this type of leukemia, the behavior of CML stem cells is very different from that of HSCs. There is substantial natural variation in lineage options within HSCs and, for CML LSCs, there is an intrinsic stability regarding neutrophil fate during the chronic disease phase. Brown’s review [3] provides evidence that the cell-of-origin for CML is an HSC. The manuscript discusses the predominance of neutrophils and mild anemia in CML, as well as the role of the *BCR-ABLp210* fusion gene as a hallmark oncogene in CML. Findings suggest that BCR-ABLp210 can direct hematopoietic stem and progenitor cells toward a myeloid fate. The author also highlights the importance of epigenetic changes in CML and the deregulation of cell fate choices in LSCs. However, the precise mechanisms underlying the abundant production of neutrophils in CML are still not fully understood.

In many cases, cancers arise from the transformation of a stem/progenitor cell that gives rise to tissue cells. At least two genetic insults or genetic alterations are needed for cancer. The first event gives rise to a cancer-initiating cell (a preleukemic cell in the case of leukemia) that remains dormant because these cells are found in persons who will never develop cancer. The second insult transforms the cancer-initiating cell into a CSC that is required for cancer. This process is perfectly exemplified by most cases of childhood B-Cell leukemia. Modelling of the disease in vivo is essential to fully understand this process. Isidro-Hernández et al. [4] focus on childhood leukemogenesis and revise a previously proposed “multi-step” or “multi-hit” mechanism that involves both in utero and postnatal steps. The authors discuss the high frequency of preleukemic “hits” compared to the incidence of childhood leukemia itself, highlighting the need to understand why only a small percentage of children with preleukemic hits develop full-blown leukemia. The study emphasizes the importance of mouse models that recapitulate the multistage process of childhood B-cell acute lymphoblastic leukemia (B-ALL) in order to identify the environmental and genetic factors associated with increased disease risk. The authors suggest that these mouse models will be essential to uncovering the factors that are directly linked to the increased risk of childhood leukemia.

The development of reliable cancer stem cell models provides valuable experimental approaches, not only to understanding the disease, but also to identifying novel testable therapeutic alternatives to therapy-resistant cancer. Telang’s review [5] focuses on the development of stem cell models to study therapy-resistant cancer, particularly breast and colon cancer. The author highlights the emergence of drug-resistant tumor-initiating CSC populations as a major cause of therapy resistance and metastatic progression. The study discusses the activation of specific cancer-cell-signaling pathways and epithelial–mesenchymal transition in premalignant stem cells, leading to the development of a metastatic phenotype. The review also describes the development of drug-resistant stem cell models for different molecular subtypes of breast cancer and genetically predisposed colon cancer. These models enable the examination of stem-cell-targeted growth inhibitory efficacy using naturally occurring dietary phytochemicals. The study emphasizes the importance of these models in identifying novel therapeutic alternatives for therapy-resistant breast and colon cancer.

In this regard, the elimination of CSCs is crucial to avoid chemoresistance, and the recurrence and metastasis of cancer. The targeting of CSCs by differentiation therapy is a research area that relies on the terminal differentiation of cancer cells until they are unable to divide, and lose tumorigenicity as growth and metastatic ability are impeded. Mehus et al. [6] investigate the potential of pevonedistat (PVD), a neddylation inhibitor, to inhibit the expression of SOX2 and reduce stemness in arsenite-transformed urothelial cells. The results demonstrate that PVD treatment causes morphological changes, reduces cell growth, attenuates sphere formation, induces apoptosis, and elevates the expression of terminal differentiation markers. When combined with cisplatin, PVD further increases the expression of terminal differentiation markers and leads to increased cell death. These findings suggest that PVD, either alone or in combination with cisplatin, could be a potential differentiation therapy or alternative treatment for muscle invasive urothelial cancer that is resistant to cisplatin.

The overexpression of retinoic acid receptor γ (RARγ) and its activation plays a role in the abnormal behavior of CSCs for several cancers. CSCs express RARγ for maintenance; therefore, RARγ provides an interesting target to fight against them. The review by Brown [7] highlights the role of the retinoic acid pathway in cancer development and its potential as a therapeutic target. The summary discusses the deregulation of cytosolic aldehyde dehydrogenases, which mediate the synthesis of all-*trans* retinoic acid, in various human cancers. Inhibiting these enzymes has shown promising results in reducing cancer cell proliferation, inducing apoptosis, and sensitizing cancer cells to chemotherapeutic agents. The study also explores the role of retinoic acid receptors and RARγ as oncogenes in different types of cancer. Furthermore, the inhibition of RARγ has been shown to induce necroptosis in CSC, suggesting that targeting the retinoic acid pathway holds promise for the development of new drugs to eradicate CSC.

In this Special Issue, Azagra et al. [8] discuss the role of NSD2, a member of the Nuclear SET Domain (NSD) family, in epigenetic modifications and its relevance in hematological disorders. The study highlights the importance of H3K36 dimethylation, which is primarily carried out by NSD family proteins, in genome regulation. NSD2 is frequently altered in various tumors, especially hematological malignancies. The authors review recent findings on the development of new compounds that target the oncogenic forms of NSD2. The study suggests that NSD2 could be a promising target for therapeutic interventions in hematological disorders, and further research is needed to explore the NSD2’s potential as an anticancer candidate.

CSC research continues to provide valuable insights into the mechanisms of tumor initiation, progression, and therapy resistance. The papers included in this Special Issue discuss promising therapeutic strategies, including the use of specific inhibitors, the targeting of signaling pathways, and the development of reliable stem cell models. By advancing our understanding of CSCs and their unique properties, we can pave the way for the development of novel and more effective treatments for various types of cancer. Further research and collaborations are necessary to translate these findings into clinical applications and ultimately improve patient outcomes in the battle against cancer.
